# Malakit: an innovative pilot project to self-diagnose and self-treat malaria among illegal gold miners in the Guiana Shield

**DOI:** 10.1186/s12936-018-2306-5

**Published:** 2018-04-10

**Authors:** Maylis Douine, Alice Sanna, Muriel Galindo, Lise Musset, Vincent Pommier de Santi, Paola Marchesini, Edgard Dias Magalhaes, Martha Suarez-Mutis, Helene Hiwat, Mathieu Nacher, Stephen Vreden, Laure Garancher

**Affiliations:** 1 Centre d’Investigation Clinique Antilles-Guyane (Inserm 1424), Cayenne Hospital, Avenue des Flamboyant, BP 6006, Cayenne Cedex, 97306 French Guiana France; 2 Health Regional Agency, Cayenne, French Guiana France; 3 Laboratoire de Parasitologie, Centre National de Référence du Paludisme, Institut Pasteur de la Guyane, Cayenne, French Guiana France; 4 French Armed Forces Center for Epidemiology and Public Health (CESPA), Camp Militaire de Sainte Marthe, Marseille, France; 5 IRD, AP-HM, VITROME, SSA, IHU-Méditerranée Infection, Aix Marseille Univ, Marseille, France; 6 National Malaria Control Programme, Ministry of Health, Brasilia, Brazil; 7 International Affairs Office, Ministry of Health, Brasilia, Brazil; 8 Laboratory of Parasitology, Institute Oswaldo Cruz, Rio de Janeiro, Brazil; 9 National Malaria Programme, Ministry of Health, Paramaribo, Suriname; 10 Epidemiology of Tropical Parasitoses, EA 3593, Université de Guyane, Cayenne, French Guiana France; 11 Foundation for Scientific Research Suriname (SWOS), Paramaribo, Suriname; 12 Pan American Health Organization, Barbados Office, Bridgetown, Barbados

**Keywords:** Malaria, Control strategies, Gold mining, Mobile population, Cross-border

## Abstract

**Background:**

Illegal gold miners in French Guiana, a French overseas territory (‘département’) located in Amazonia, often carry malaria parasites (up to 46.8%). While the Guiana Shield Region aims at malaria elimination, the high prevalence of *Plasmodium* in this hard-to-reach population in conjunction with frequent incorrect use of artemisinin-based anti-malarials could favour the emergence of resistant parasites. Due to geographical and regulatory issues in French Guiana, usual malaria control strategies cannot be implemented in this particular context. Therefore, new strategies targeting this specific population in the forest are required.

**Methods:**

Numerous discussions among health institutions and scientific partners from French Guiana, Brazil and Suriname have led to an innovative project based on the distribution of kits for self-diagnosis and self-treatment of *Plasmodium* infections. The kit-distribution will be implemented at “resting sites”, which are areas across the border of French Guiana regularly frequented by gold miners. The main objective is to increase the appropriate use and complete malaria treatment after a positive malaria diagnosis with a rapid test, which will be evaluated with before-and-after cross-sectional studies. Monitoring indicators will be collected from health mediators at the time of kit distribution and during subsequent visits, and from illegal gold miners themselves, through a smartphone application. The project funding is multisource, including Ministries of Health of the three countries, WHO/PAHO, and the European Union.

**Results:**

This project will start in April 2018 as a 18 month pilot study led by the Clinical Investigation Centre of Cayenne. Results should be available at the end of 2019.

**Discussion:**

This innovative approach may have several limitations which should be taken into account, as potential side effects, kit misuse or resale, declarative main criteria, or no *Plasmodium vivax* curative treatment. Close monitoring is thus needed.

**Conclusions:**

This project may be the best available solution to a specific and important public health challenge in the Guiana Shield. If the use of self-diagnosis and self-treatment approach is effective, this strategy could be sustained by health institutions in the region.

## Background

### Context of malaria in illegal gold mines in French Guiana

Exceptional situations call for exceptional responses. This is the case for the malaria situation in French Guiana, a French territory located in the Amazonian forest between Suriname and Brazil. Although malaria is under control in the local population [1, 2], illegal gold miners working deep in the rainforest are massively infected. Studies have shown that malaria prevalence in this population is very high, ranging from 3.6 to 46.8% [3, 4]. Many infected miners are asymptomatic (40–84%), thus constituting a huge *Plasmodium* reservoir, with a majority of *Plasmodium falciparum* (58.5–70.7%) [3, 4]. The remoteness of the mining camp and the illegal administrative situation of these people lead to frequent self-medication. A study showed that 52% of gold miners self-medicated during their last malaria crisis, using under-the-counter drugs and with poor adherence (40%) [5]. The main anti-malarial drug used for malaria-like symptoms is Artecom^®^ (90%), which contains dihydroartemisinin (32 mg) associated with piperaquine (320 mg) and trimethoprim (90 mg) and a single dose of primaquine. The quantity of primaquine is not mentioned on the packaging, which does not respect pharmaceutical standards [6]. This drug is not registered with a stringent medicine regulatory authority or the WHO prequalified programme, thus it is illegal in French Guiana and neighbouring countries.

Several studies have clearly identified a risk of emergence of artemisinin resistance due to high malaria endemicity and inappropriate self-medication with artemisinin-based combination therapy (ACT) among gold miners in French Guiana [4, 5]. Illegal gold miners are mobile populations mainly originating from the poorest states of Brazil [7]. They could contribute to malaria reintroduction in transmission-free areas [8]. Hence Surinamese authorities declare that the majority of malaria cases diagnosed in Suriname are imported from French Guiana by miners, jeopardizing the successful national programme to eliminate malaria in the Surinamese population [9–11]. In Brazil, malaria cases from French Guiana represent 21% of the imported cases [12]. Therefore, there is an urgent need to act among this specific population to avoid the emergence of artemisinin resistant *P. falciparum* parasites and to facilitate malaria elimination on the Guiana Shield.

### A need for new adapted strategies

The usual malaria control strategies [13] cannot be implemented in this context of undocumented persons living in French Guiana, hidden deep in the rainforest where they conduct illegal activities. The remoteness of mining camps (sometimes 4–5 days by canoe and by foot), the high number of mining sites (several hundred), the high mobility of gold miners and the insecurity hamper direct action at mining sites [4, 7]. Moreover, French regulations do not allow people who are not health professionals to carry out malaria diagnostic tests and to deliver treatment. Therefore, other public health initiatives that have been designed for hard-to-reach people living in border areas, such as deploying health agents or mass drug administration, cannot be implemented in this French territory. New strategies targeting populations in the French Guianese forest are required, complementing malaria care already implemented at resting sites by the malaria programmes in Suriname and Brazil.

## Methods

### A long process of political discussions

Several strategies have been discussed over the past few years at different levels: (1) local, with the Regional Health Agency (RHA), regional state representatives, researchers, French military health service and health care structures; (2) national, with the Ministry of Health, the Ministry of Foreign Affairs, Home Affairs, Ministry of Overseas Territories, Ministry of defense; (3) international, with representatives of Brazil (both federal by Amapa state and municipality level) and Suriname, World/Pan-American Health Organization (WHO, PAHO), and the Global Fund.

### Malakit project

These discussions led to a new innovative approach called “Malakit”, based on what has been learnt from previous studies on mobility, knowledge, attitudes and behaviour among this miner population [4, 5]. This research-action project links scientific and operational partners in a common public health intervention. It is based on the distribution of kits for self-diagnosis and self-treatment against *Plasmodium* infections in cross-border areas, after training of the target population by health mediators and in the subsequent evaluation by a before-and-after design. The first objective of the project is to increase the use of an appropriate and complete malaria treatment (approved ACT + single-dose of primaquine [14]), after a malaria diagnosis with a rapid diagnostic test (RDT). The secondary objectives are to reduce the malaria prevalence among illegal gold miners, and to improve their knowledge about malaria and its prevention.

**Fig. 1:**
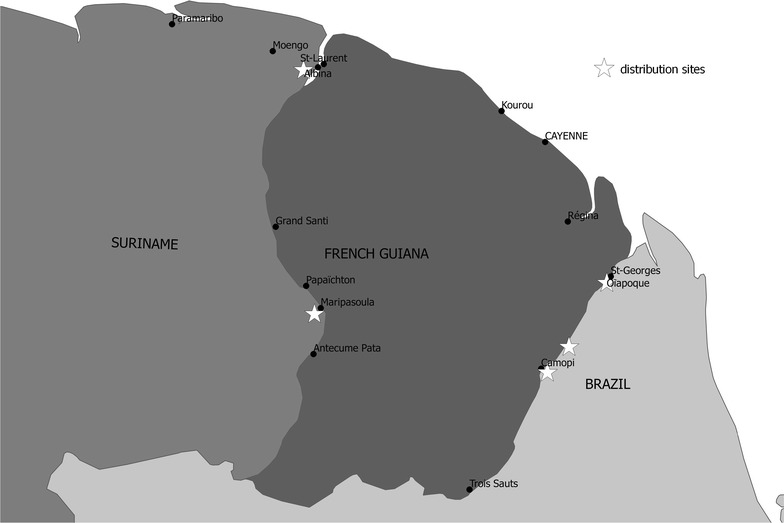
Distribution sites for the Malakit project

**Fig. 2:**
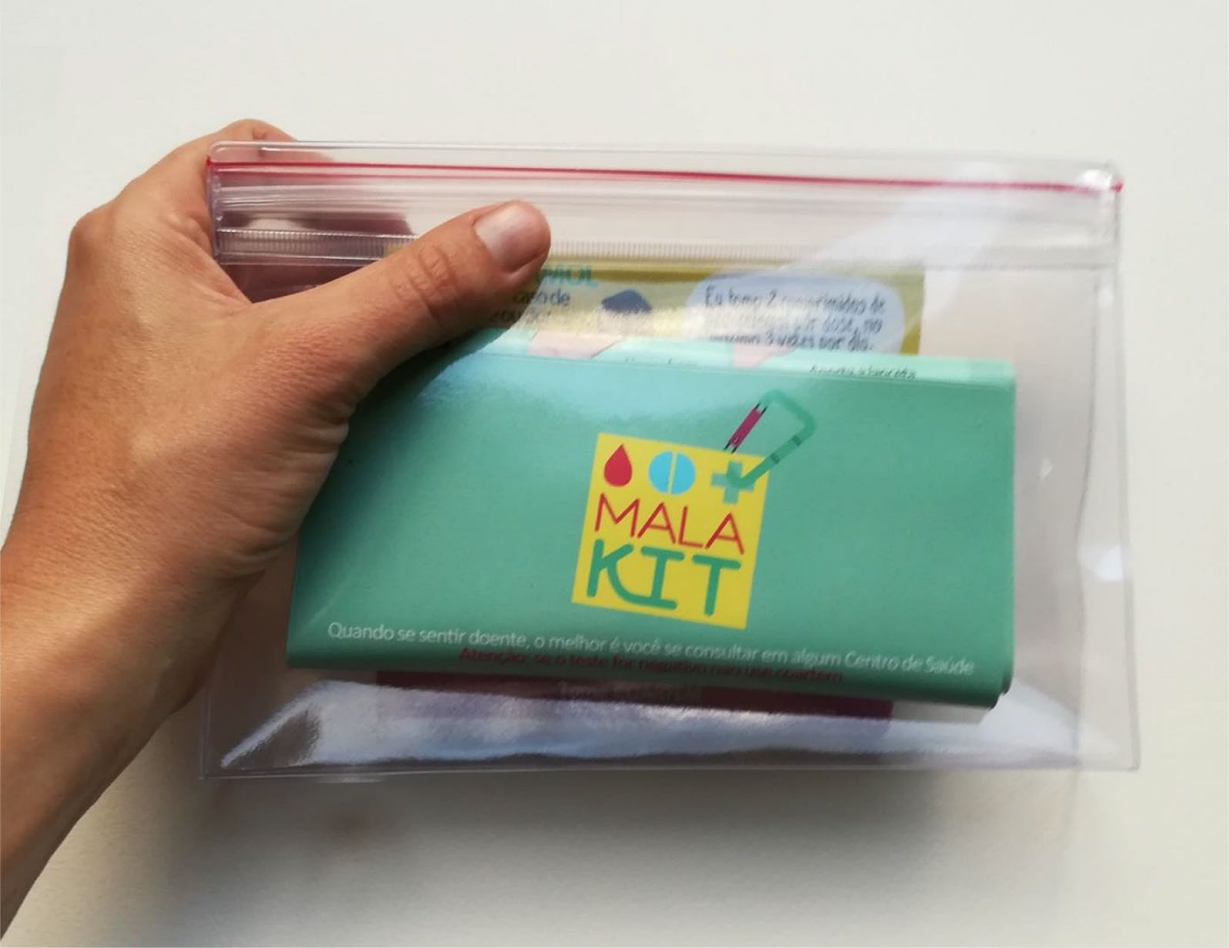
Self-diagnosis self-treatment kits

The kit-distribution will be implemented at “resting sites”, which are transborder areas located on the Surinamese side of the Maroni river and on the Brazilian side of the Oyapock river (Fig. 1). Gold miners go to these informal settlements for transactions of gold and logistical supplies. A previous study along the Maroni river demonstrated that these sites are strategic to efficiently target this highly mobile population for public health actions [7]. As sickness hampers working and thus the capacity to earn money, gold miners are interested in health promotion activities. The kits will contain three RDTs and one complete ACT/single-dose primaquine (15 mg) treatment (Fig. 2). A strip of acetaminophen tablets will also be included in order to offer an alternative if the RDT is negative while the miner experiences fever. The RDT was chosen according to the following criteria: (1) ease of use: result of the test in two bands, positive or negative, because the target population is often poorly educated; (2) the individual packaging of the test and its buffer; (3) stability at temperature higher than 35 °C; (4) a shelf life of at least 12 months; (5) the CE (European Community) label and the WHO pre-qualification. Only one RDT candidate met all of these requirements, the ACCESSBIO’s CareStart™ Malaria pLDH (Pan) test. The selected ACT, artemether 20 mg/lumefantrine 120 mg, is the one recommended in the three countries involved in the project according to WHO recommendations [15]. It is efficient against *P. falciparum* and *Plasmodium vivax*, the two most frequent malaria parasite species circulating in French Guiana. Kit users will be instructed that in case of a new malaria episode within a month after a first attack, they should go to a health centre to be checked and eventually treated for *P. vivax* relapse.

### Kit distribution and training

The target population will be informed about the project by word of mouth and through an animated video to share on social networks (such as whatsapp or facebook, which are widely used by gold miners), showing through a short story the objectives of the project and where to get the kits. Inclusion criteria are: agreeing to participate in the study, being over 15 years old—with parental authorization for minors between 15 and 17 years old—, having succeeded to correctly perform a self-RDT and having well understood treatment instructions. Six health mediators, employed for the project by local associations, will be trained on malaria, limitations and risks of kit utilization and research concepts, to be able to perform the training of gold miners to use the kits. Teaching material will include demonstration, practice, video and illustrated instructions. Training will contain information about severity symptoms (drowsiness, convulsions, absence of urine, breathing difficulty, jaundiced eyes), potential interactions with cardiac treatments, and the insufficiency of drug absorption in case of vomiting. In those cases, participants will be invited to start the treatment and rapidly consult at a health centre. The clinical signs have been defined by the Malakit scientific committee in a way that should easily be recognizable by participants. Women will be informed that in case of pregnancy, they should not take primaquine, and that a medical consultation is recommended.

**Fig. 3:**
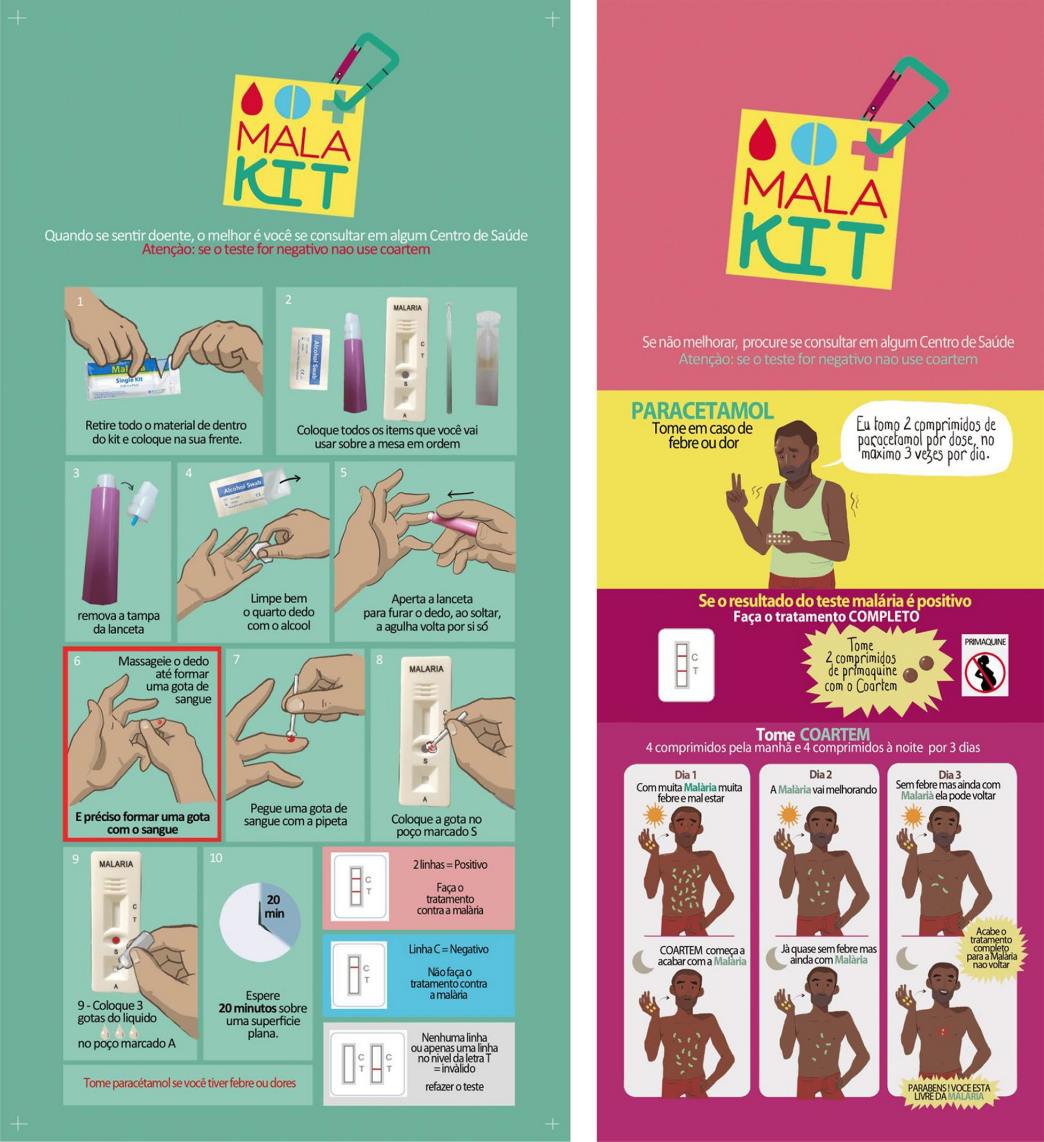
Illustrated instructions on the kits

Each participant will receive one kit for personal use and a long-lasting impregnated mosquito net. Gold miners may replenish their kits—and get refresher training if necessary—during their following visits to the resting sites participating to the study. Because of the frequent illiteracy of the target population, most of the tools used for training and explanations printed on the kits are based on drawings, videos, and pictograms (Fig. 3). They will be in Portuguese as almost all the target population is Brazilian. The few non-Brazilian people speak or at least understand Portuguese, which is the « forest language » [7].

### Evaluation of the intervention

The main evaluation criteria of the intervention will be the appropriate use of the malaria treatment (a complete treatment course in case of a positive testing). Secondary criteria will be: (1) the PCR-*Plasmodium* prevalence among the miner population; (2) the use of preventive measures; and (3) malaria knowledge. On the Surinamese border, a first cross-sectional survey was performed in 2015 to evaluate these criteria (methodology described in Ref. [3]). On the Brazilian border, the same survey will be implemented during the beginning of the kit distribution. After 1 year of kit distribution, a new cross-sectional study will be carried out on the Brazilian and Surinamese border.

Monitoring indicators will be collected from health mediators and illegal gold miners themselves throughout the study. Health mediators will collect socio-demographic and professional life data at the first visit and data about the utilization of the kit at subsequent visits. The participant has the right to not answer all questions. No identifying data will be collected: for example the place of work will be “basin river of mining” instead of the precise mining site. A smartphone application will be developed and made available to participants of the study. Field missions showed that about 80% of the target population has a smartphone and that they were very enthusiastic about the concept. This app will contain an information module with a reminder video on how to use the kit and an interactive module for registering date of fever, RDT results, and treatment adherence. In case of a positive RDT, a reminder phone alert will help people to take the full treatment course. Data from the app will be sent automatically to the server each time the participant gets a connection.

All data will be collected and directly anonymized. A correspondence list between name, (declarative name, no identity card requested), date of birth and anonymous number will be kept on a secure server. This list will be only used by the investigators to identify persons coming back at a distribution site who have lost the kit and its anonymous number, and to erase data from people who change their mind and do not want to be part of the study anymore.

### Regulatory context

Written informed consent will be collected for each participant. Ethical committee authorization from Suriname and Brazil are requested as inclusions will take place on their territory. In France, the use of self-medication is permitted for oneself, but it is not permitted to give the treatment for someone else. This is why the project targets the population who are able to perform a RDT and to take the treatment by themself: persons over 15 years. Thus children cannot be included in the project. The training will emphasize that children should go to a health center quickly as soon as they are sick. Moreover, the evaluation of the project is an individual evaluation of the capacity to self-diagnose and self-treat malaria. Therefore, the data should be collected at an individual scale. An independent “Data Monitoring Safety Board” will regularly check the data to monitor the potential adverse effects of the project.

### Governance

**Fig. 4:**
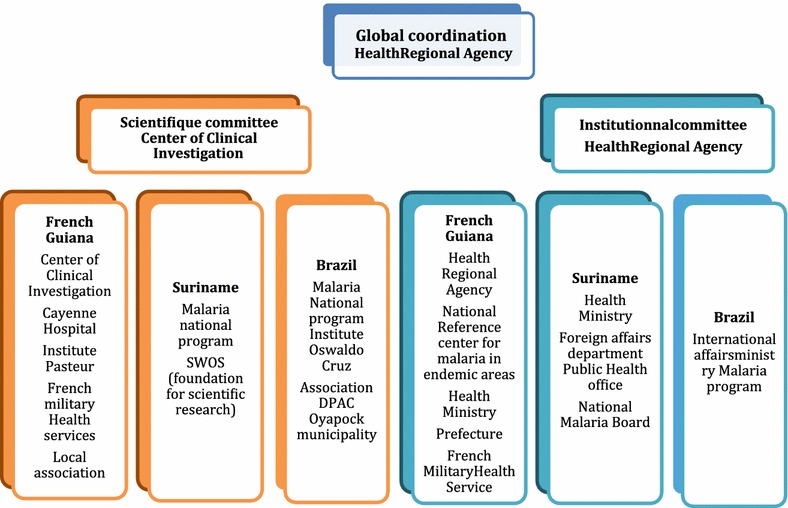
Organization chart of the project

An institutional committee has been created as well as a scientific committee under the coordination of the RHA (Fig. 4).

## Results

This project will be launched in April 2018 as a pilot study by the Clinical Investigation Centre of Cayenne, which will oversee the implementation and the monitoring-evaluation. Monitoring data will be collected during 1 year and cross-sectional studies will be conducted before/after the distribution on both borders. Data will be shared with investigators and the malaria programme of each country all along the project. Based on the final evaluation, the health authorities will consider the possibility of a long-term intervention. Results should be available at the end of 2019.

## Discussion

Although there are examples of presumptive treatment in case of malaria symptoms in some particular situations (standby emergency treatment in travelers or home-based management of malaria), the strategy presented here is innovative because it is a large scale global and integrative approach in a specific population, aiming to avoid presumptive or erratic treatment and promoting the correct treatment after a malaria diagnosis.

This pilot phase needs a close monitoring (from investigators and the DSMB) to evaluate potential kit misuse (children, pregnant women), incorrect RDT handling and/or interpretation, or treatment side effect. Several limitations of this project should be taken into account. Kits may be sold, especially at the beginning of the project. Since “anything rare is expensive”, a large kit diffusion should avoid parallel market. Instructions are printed directly on the kit to ensure good utilization, even without the training. Data about kit origin and price, if it was purchased, will be collected by facilitators to check this aspect. The project does not include species diagnosis thus no curative treatment for *P. vivax* because the public health challenges of this species are less important. However, information will be given to the participants about potential relapses. This project targets symptomatic malaria attacks, considering that the parasitic load is significantly higher in symptomatic persons than in asymptomatic carriers [16]. Thus with an early diagnosis and treatment including a single-dose of primaquine, *Plasmodium* transmission should decrease, as shown in many other malaria control projects, notably in neighbouring Suriname [11]. The final evaluation is based on a declarative criteria, self-reported kit utilization (self-declaration through phone app and hetero-declaration through facilitators), leading to a potential declarative bias toward the desired answer. However, because this approach with self-treatment at a large scale is new, the individual evaluation of behaviour improvement in case of malaria symptoms is essential. But even if there is an improvement of malaria treatment use, resistant parasites could emerge at the same time. Samples collected during the evaluation will be sent to the National Malaria Reference Centre at the Institut Pasteur de la Guyane for the surveillance of resistance.

## Conclusions

This innovative project aims to improve the correct use of malaria treatment in order to avoid artemisinin resistance selection in the very specific and cross-border context of the Amazonian forest. The future policy decision to prolong the intervention based on the evaluation of the pilot intervention will be carried out carefully, minding potential biases linked with the before-and-after design and with the declarative nature of several indicators. Nevertheless, in the context of the current French legal framework and hard-to-reach target population, this project may be the best available solution to a complex and important public health challenge. If the use of self-diagnosis and self-treatment approach is effective, this strategy could be extended to other parts of the world facing similar contexts.

## Abbreviations

ACT: artemisinin-based combination therapy
CE: European Community
RDT: rapid diagnostic test
RHA: Regional Health Agency
WHO/PAHO: World Health Organization/Pan-American Health Organization
